# Short-term effects of bone meal powder on soil phosphorus availability, seed germination, and early growth parameters of Malabar Spinach (*Basella alba*)

**DOI:** 10.1371/journal.pone.0351758

**Published:** 2026-06-18

**Authors:** Aishwa Roy, Protima Sarker

**Affiliations:** Department of Environmental Science, Faculty of Science, Noakhali Science and Technology University, Noakhali, Bangladesh; VIT University, INDIA

## Abstract

There is a growing need to find alternatives to chemical fertilizers, particularly for Phosphorus supply. Chemical fertilizers contribute to environmental problems and natural phosphate resources are depleting. The main objective of the study was to examine the effect of bone meal powder (BMP) as an organic fertilizer on soil phosphorus availability, seed germination, and early growth performance of Malabar spinach (*Basella alba*). Two experiments were conducted: soil chemical analysis and pot experiment. Experiment 1 referred to highly saline soil-High Electrical Conductivity (EC) and Experiment 2 used non-saline- Low Electrical Conductivity (EC) soil. The treatments included: T1: High EC soil; only potting media; no BMP, T2: High EC soil amended with BMP, T3: Low EC soil; only potting media; no BMP, T4: Low EC Soil amended with BMP. All treatments were used to analyze the soil chemical properties, including soil pH, EC and Phosphorus. For the pot experiment, treatments (T3 and T4) were combined in a completely randomized design with three replicates and the data were collected on germination and early growth parameters of malabar spinach. Results revealed that the application of BMP increased soil pH, EC and notably improved phosphorus availability, compared to the control in both soils. However, BMP negatively affected germination and early growth variables in experimental soil 2. In spite of the great differences in shoot length were observed in the application of BMP, only minor differences were found in root lengths and fresh weight. The correlation analysis showed that mean germination time had negative relationships with growth parameters. The findings suggest that BMP may have potential as an inexpensive and eco-friendly fertilizer, although further large-scale field experiments are necessary to confirm its effectiveness. These results may inform researchers, stakeholders, and agricultural practitioners regarding the potential use of BMP.

## Introduction

Over the past 120 years, the global population has risen from 1.65 billion to 7.7 billion and is projected to reach 9.7 billion by 2050. As a result, according to the IPCC report, global temperatures are projected to rise by 1.5°C above preindustrial levels between 2030 and 2052. Ultimately, global food demand is also predicted to increase by 70% due to current and future climate change [1,2]. Several studies have examined the use of both mineral and organic fertilizers to restore soil fertility [3]. Although synthesized chemical fertilizers confirm food security, they cause physicochemical and biological alterations of the soil, soil deterioration through nutrient leaching, desertification, declining crop yields, greenhouse gas emissions, and groundwater contamination [2,4,5]. Since agriculture in Bangladesh relies heavily on chemical fertilizers to meet the high food demand, it is facing these problems.. The challenges posed by the chemical fertilizers in Bangladesh emphasize the need for research on using organic fertilizer as an alternative in plant growth. Modern agricultural technologies are improving global food supply by implementing sustainable agronomic practices [2,5]. Different types of natural fertilizers, produced from animal manure, crop wastes, etc. or agricultural practices like waste compost amendments, fallows, and green manure can be effectively incorporated in alignment with the principles of sustainable development [3]. Unlike mineral fertilizers, the effects of organic fertilizers are long-lasting since mineralization happens gradually, which means that they benefit plants with prolonged periods of growth and is only apparent in the years after they are used [6]. The results in the study of Mohammed et al. [7] indicate that organic fertilizers significantly increase yields.

Bone meal powder, a common organic fertilizer derived from ground animal bones, contains various nutrients and organic compounds essential for plant growth [8]. It is high in nitrogen, phosphorus, and calcium [9]. Phosphorus is a primary nutrient for plants in the soil [10]. Animal Meat Bone Meal (MBM) can serve as an effective alternative to inorganic fertilizers, as field tests in Europe have demonstrated its high fertilization value for crop production [6]. The availability of rock phosphate is projected to be endangered as the global demand for P is predicted to increase by 2050 [11]. As P utilization should be efficiently managed for sustainable agricultural ecosystems, it can be recovered by using organic waste disposal such as- bone waste as a sustainable and alternative P fertilizer towards an efficient P cycle [11]. MBM is a by-product of the rendering industry and considered a viable nutrient supplement for agricultural systems because of its high P concentrations, particularly for organic farms that concentrate in plant production. There are above 18 million tons of MBM production in the European Union in a year [6]. MBM consists of Biological apatite which is an inorganic calcium phosphate salt, results in higher solubility compared to geological apatite- Hydroxyapatite (HA) [11,12]. The study of Zimmer et al. examined the effect of bonemeal in soil properties and growth of *Pelargonium graveolens* [12]. While Załuszniewska et al. emphasized the effect of same amendment on phosphorus content and uptake by different crops, soil available P balance and soil pH [13]. The study by Pan et al. confirms that bonemeal was regarded as the most effective fertilizer among other organic and inorganic fertilizers with highest germination shown in tomato and cucumber crops [14]. Furthermore, another study assessed the impacts of bonemeal powder on growth and yield or development [14].

In the present study, Malabar spinach was grown as a test seedling, particularly due to its rapid, vigorous growth and adaptability to warm conditions [15], and its common cultivation in Bangladesh. This study evaluated how bonemeal powder as organic fertilizer affected soil phosphorus availability, seed germination and the early growth performance of Malabar Spinach. To achieve this aim, the study assessed the effect of BMP on (1) soil properties: pH, Electrical Conductivity (EC), particularly phosphorus availability; (2) seed germination parameters of Malabar Spinach; (3) early growth parameters of Malabar Spinach.

## Materials and methods

### Experimental design

#### Collection of soil sample and bone-meal powder.

For Experiment 1 (Exp-1) the soil sample was collected from the field beside the cafeteria inside Noakhali Science and Technology University, Noakhali. For Experiment 2 (Exp-2) the soil sample was collected from an agricultural field located on the Thakkor, Noakhali Science and Technology University Road. The trowel is used to sample intact soil from a depth of 0–15 cm at four corners. The bonemeal powder was purchased from a nursery. No specific permits were required for soil collection because the sampling sites were located in non-protected areas and the study did not involve endangered or protected species. The ethical approval and informed consent were not required.

#### Soil preparation.

The collected soil sample was air dried by spreading thinly on a plastic bag. The sample was dried for 1 week. Afterwards, it was sieved through a 0.5 mm sieve for laboratory analysis of the soil sample.

#### Soil chemical analysis.

Soil pH, electrical conductivity (EC), and available phosphorus (Olsen-P) were determined for the highly saline soil (high EC) used in Experiment 1 and non-saline soil (low EC) in Experiment 2. The analysis was conducted for four treatments: T1: High EC soil; only potting media; no BMP (Control 1), T2: High EC soil amended with BMP (Control 1 + BMP), T3: Low EC soil; only potting media; no BMP (Control 2), T4: Low EC Soil amended with BMP (Control 2 + BMP). The study evaluated the effect of BMP application on soil chemical properties, particularly phosphorus availability. 1.5g of BMP was mixed separately with each soil.

#### Pot trial.

The pot experiment (Experiment 2) was conducted in a completely randomized design (CRD) [16], which used low EC soil and included two treatments (T3 and T4) with three replications each. A total 6 pots (diameter: 4 cm) were used and each pot contained 200g soil. Replication labels include- T3: T_3.1_, T_3.2_, T_3.3_; T4: T_4.1_, T_4.2_, T_4.3_.BMP as the fertilizers were mixed at the recommended rate into the soils and the pots were kept bare for 2 weeks and watered well. The seeds were sown after being soaked in water for 24 hours. Five seeds were sown per pot at a depth of approx. 2 cm.

### Data collection

#### Germination monitoring.

Seeds were considered to have germinated when the radicle was at least 2 mm in length [15]. Germination was monitored daily from the day of sowing for a total of 11 days. Within three to four days, germination started. The observed data were then used to calculate germination parameters.

#### Seedling sampling.

Data were collected at the early seedling stage immediately after the completion of the germination monitoring period. One healthy seedling was randomly selected from each pot for measurement.

#### Measurement of seedling early-growth parameters.

**Fig 1:**
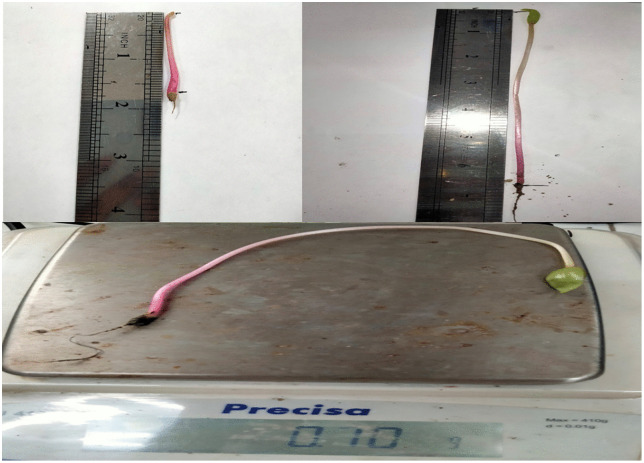
Measurement of shoot and root length, fresh weight of seedlings.

Seedling growth was assessed using the following parameters: shoot length, root length, and fresh weight. These measurements were taken to evaluate early-stage seedling performance under experimental soil 2 condition. In order to find the effect of fertilizers on seedling growth, the growth parameters such as shoot length, root length, fresh weight per seedling were measured after 11 days (Fig 1).

The average root and shoot length (inch) were measured using a measuring scale from the root level to the tip of the plant. Root length was measured using a measuring scale from root level to the tip of the longest root at harvest, and their average value was taken as the root length in inch. Harvest of one seedling from each pot, fresh weight of the whole plant was taken by an electrical balance, and their mean value was calculated as fresh weight expressed in gm/seedling.

#### Laboratory analysis of soil and BMP.

Some of the physiochemical analyses of soil were carried out in the laboratory of Soil Resource Development Institute (SRDI), Gabua, Noakhali during the period of May 2024. Some of the analyses of soil and measurement of seedling was carried out in the Soil and Environment Laboratory of Bangladesh Council of Scientific and Industrial Research (BCSIR) during the period of June 2024.

Chemical properties of the soil samples and BMP (Table 1) were determined as follows: The pH was measured in water extracts with standard electrodes [17]. The Electrical Conductivity (EC) was determined by the saturation extract method [18]. The available phosphorus (P) was determined by Olsen method [17], also known as Olsen P test in soil and by Molybdovanadate method (yellow method) [19] in Bonemeal powder (BMP). In the determination of organic carbon and organic matter, wet combustion or wet oxidation method was used [17]. Potassium of soil and Bonemeal powder was determined by flame photometer method [17]. Total nitrogen was determined by Kjeldahl method [17]. Calcium and magnesium were analysed by atomic absorption spectrophotometry method [17].

**Table 1:** Chemical Properties of control soil 1 (High EC) in Experiment 1; control soil 2 (Low EC) in Experiment 2 & Bone-meal powder (BMP).

Properties	Soil 1(Exp 1)	Soil 2(Exp-2)	BMP
**pH**	6.92	7.25	/
**EC (ds/m)**	40.6	1.08	/
**P (ppm)**	5.29	9.31	8.23
**TN (%)**	0.10	0.05	1.18
**K (meq/100g)**	0.44	0.36	0.07
**TOC/ TC (%)**	/	/	13.26
**TOM (%)**	2.04	1.12	22.86
**Ca (%)**	/	/	11.04
**Mg (%)**	/	/	2.36

N.B. BMP = Bonemeal Powder, EC = Electrical Conductivity,/ represents not determined, TP = total phosphorus, TN = total nitrogen, K = potassium, S = sulfur TOC/TC/TOM = total organic carbon/ total carbon/ total organic matter, Ca = calcium, Mg = magnesium.

### Statistical analysis

#### Germination indices.

The timings of seed germination in pots were observed carefully in order to find the effect of fertilizer on seed germination. After collecting the data, statistical analysis was performed using Microsoft Excel. Graphical representations were also prepared using the same software.

The germination parameters- First Day of Germination (FDG), Last Day of Germination (LDG), Time Spread of Germination (TSG), Germination Percentage (GP), Mean Germination Time (MGT), Mean Germination Rate (MGR), and Germination Index (GI) were calculated using following formulas based on previous scholar’s work [20–23].


$$\begin{array}{c} \text{First Day of Germination }(\text{FDG})\;=\text{ Day on which the first germination event occurred} \end{array}$$

$$\begin{array}{c} \text{Last Day of Germination }(\text{LDG})\;=\text{ Day on which the last germination event occurred} \end{array}$$

$$\begin{array}{c} \text{Time Spread of Germination }(\text{TSG})\;=\text{ The time in days between the first and last germination events occurring in a seed lot} \end{array}$$

$$\begin{array}{c} \text{Germinated percentage }(\text{GP})\;=(\text{SNG}/\text{SN0})\times \text{100} \end{array}$$

where, “*SNG*” represents germinated seeds in total and “*SN0*” represents total number of viable seeds or total sown seeds [21,23].


$$\begin{array}{c} \text{Germination Index }(\text{GI})=\sum \frac{number\;of\;germinated\;seeds}{day\;when\;the\;seed\;germinated} \end{array}$$

$$\begin{array}{c} \text{The Mean germination time }(\text{days})(\text{MGT})=\frac{\sum niti}{\sum ni} \end{array}$$

Where, *ti* is days from the start of the experiment to the *i th* observation; *ni* is the number of seeds germinated on the *i th* day (not the cumulative number) [20].

Mean germination rate (MGR) was the reciprocal of the mean germination time [20].


$$\begin{array}{c} \text{Mean germination rate }(\text{MGR})\;=\;\text{1}/\text{MGT} \end{array}$$

#### Correlation analysis.

Pearson’s correlation analysis was performed to evaluate the relationships between germination parameters and early seedling growth traits. Pearson’s correlation coefficient was generated using RStudio (version 2024.4.2.764) (S3 File).

#### Two-sample t-test.

Two-sample t-test was conducted using RStudio (version 2024.4.2.764) to assess whether bone-meal powder treatment caused significant differences in germination and early growth parameters of Malabar spinach compared with the control treatment (S6 File).

## Results and discussion

### Bone meal powder and soil characteristics

The studied soils including soil 1 had a slightly acidic pH of 6.92, an EC of 40.6 ds/m and contained an Olsen-P content of 5.29 ppm, while soil 2 had a slightly alkaline pH of 9.31, an EC of 1.08 and Olsen- p content of 9.31 ppm. The BMP treatment was characterized by P: 8.23 ppm; TN: 1.18%; K: 0.07 meq/100 g; TOC/TC: 13.26%; Ca: 11.04, Mg: 2.36. Further details on the chemical properties of the soils and the treatment are presented in Table 1.

### Effect of bonemeal powder (BMP) on soil properties

The use of BMP with control significantly increased the values in pH, EC, and P content of two experiments and they are shown in the following Table 2.

**Table 2:** Effect of Bonemeal powder (BMP) on soil pH, Electrical Conductivity (EC) and Phosphorus (P).

Experimental conditions	Treatment	pH	EC (ds/m)	P (ppm)
**Experiment 1**	Control 1- T1	6.92	40.6	5.29
	Control 1 + BMP- T2	7.02	54.25	14.06
**Experiment 2**	Control 2- T3	7.25	1.08	9.31
	Control 2 + BMP- T4	7.64	7.29	17.77

N.B. Values represent single measurements (Total sample size, N = 4; group size, n = 1 per treatment.).

Since bone meal can be used as an environmentally friendly input to improve soil physicochemical properties and boost plant growth, its use as a soil amendment is becoming a more significant and promising strategy in agriculture. Soil pH, EC, concentration of Olsen-P generally can be increased with high doses of BMP. This could be explained by the fact that bone meal powder contains significant amounts of mineral elements that increase the availability of nutrients in the soil.

The pH of the control soil and the treated soil with BMP were moderately acidic and moderately basic, respectively, with values of 6.92 and 7.02, respectively (Exp-1). In Exp 2, soil pH was the highest at 7.64 in the treated soil compared to the control soil. Deydier et al. reported a minor increase in soil pH in response to the application of Meat bone meal [24]. Atuah et al. and Asare also reported a significant increase in soil pH due to increased concentrations of bone meal [25,26]. Atuah et al. reported this is caused due to release of hydroxide and alkali ions such as Al3+ in the soil [25].

**Fig 2:**
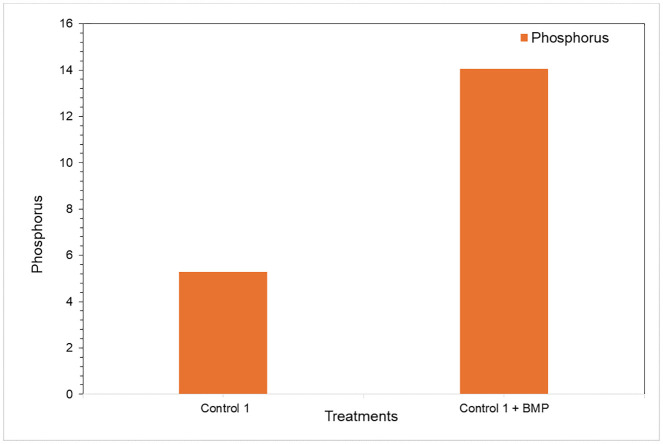
Soil phosphorus availability under control and BMP (Experiment 1).

**Fig 3:**
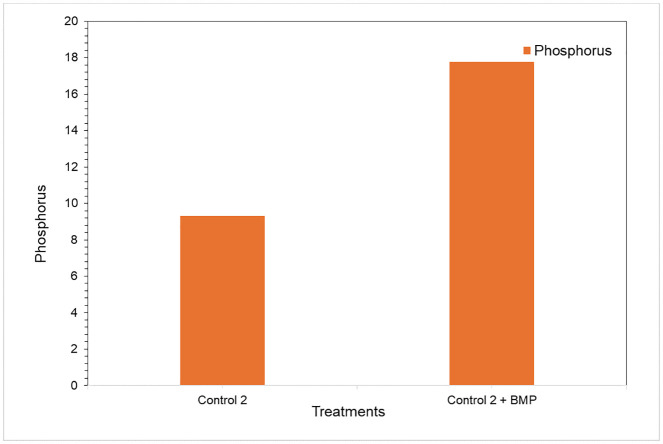
Soil phosphorus availability under control and BMP (Experiment 2).

In terms of both experiments, phosphorus was greater in the soil with BMP than in the control. The phosphorus (P) of the control was 5.29 ppm while that of the treated soil was 14.06 ppm for Exp-1. Similarly, the P of soil receiving BMP increased by 8.46 ppm in Exp-2 (Table 2, Figs 2 and 3). In the study by Liu et al. P derived from MBM was available in a more soluble form in non-calcareous soils [27]. So, BMP may release P for a long time and reduce the P transportation by runoff. In the study of Załuszniewska et al., the abundance of available P in soil increased with a rise in soil pH in Meat Bone Meal (MBM) treatments [13]. The favorable action of BM on the Olsen-P concentration in soil has been documented by many [28,29].

Furthermore, the mobility and availability of P after using BM determined by some physicochemical and mineralogical properties of boneapatite. The soil pH and phosphorus are positively related (Table 2). Soil pH rises when it interacts with phosphate derived from bonemeal and reduce exchangeable Al³⁺ in the soil. The opposite trend was noted in experiments conducted by Nogalska et al., where the available P content of soil increased with a drop in pH [29]. Slightly acidic soils are most abundant in available P. In MBM, P is present in organic form (meat fraction) and in the form of hydroxyapatite (bone fraction). The P in hydroxyapatite is released in an acidic environment and in soil colonized by mycorrhizal fungi [30] and/or P-solubilizing bacteria [31]. The P dissolves slowly after using MBM as fertilizer compared to mineral fertilizer. This acidic soil makes it easier to mobilise the poorly soluble phosphorus compounds found in bone hydroxyapatite, increasing the amount of phosphorus available for plant uptake. This is due to accumulating large amounts of P in the soil. However, the fertilizing effect of MBM is generally greater in soils characterized by a low abundance of available P. In contrast, organic P is readily available to soil for plants. Some other environmental and biological factors, including crop species, weather conditions can also increase the available P content of soil [11,32,33]. Nogalska & Zaluszniewska investigated the direct and indirect effects of increasing doses of Meat and Bone Meal (MBM) on the available phosphorus content of soil and the total phosphorus content in crops [34]. The use of MBM supplied only 20% of the phosphorus to plants in the first year, whereas 60% was still present three years [6]. Liu et al. suggested MBM as an excellent supply of phosphorus for graining crops, especially silage maize [27].

The results of both experiments indicate that the bone meal-treated soil had a higher Electrical Conductivity (EC) compared to the control where the value rose from 40.6 Ds/m to 54.25 Ds/m for Exp-1 and from 1.08 to 7.29 for Exp-2 (Table 2). According to a previous study, EC of soil increased than control after using BMP [26]. In the study, EC and P concentrations under anaerobic circumstances exhibited an extremely positive relationship [35]. Cation exchange is relatively restricted in neutral soil [32]. Since soil electrical conductivity (EC) increases, both cations and anions become more mobile. Higher EC releases more soluble nutrients and metals into the soil, along with containing inorganic components from bone meal [36]. Anions, or negatively charged ions, are more mobile than cations in alkaline soils, suggesting a higher cation exchange capacity (CEC) [32]. In the end, increased ion mobility raises soil EC overall.

### Impact of BMP on seed germination of Malabar Spinach

**Fig 4:**
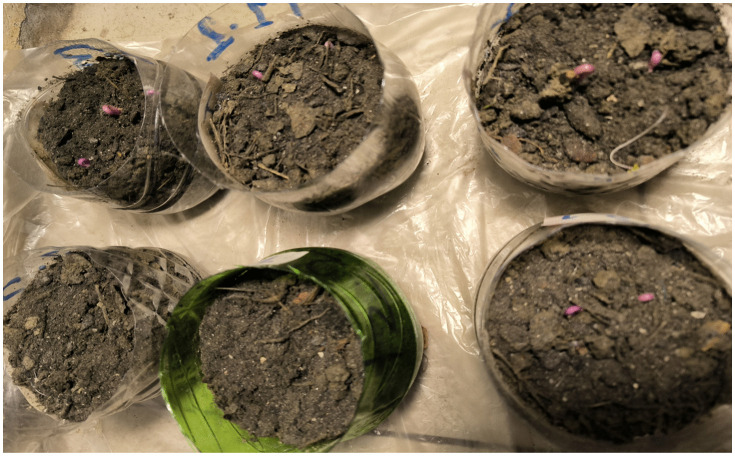
1st day of germination in 2^nd^ experiment.

**Fig 5:**
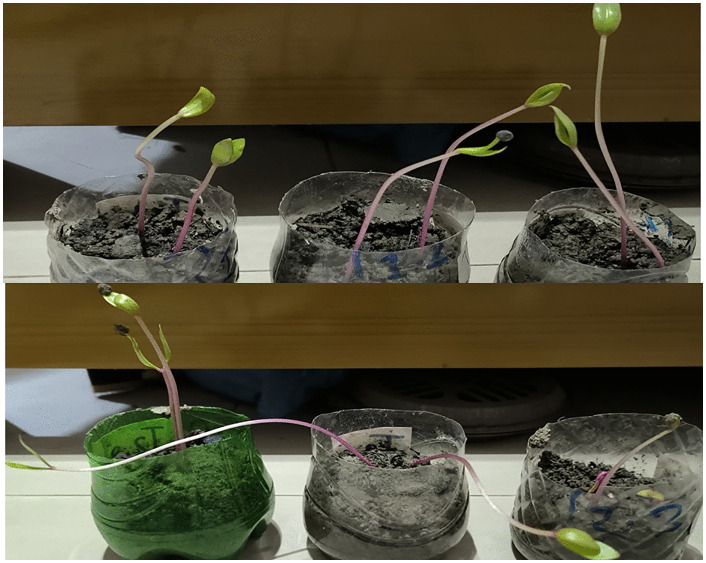
Last day of germination after 4th day of sowing and thinning in 2^nd^ experiment.

**Fig 6:**
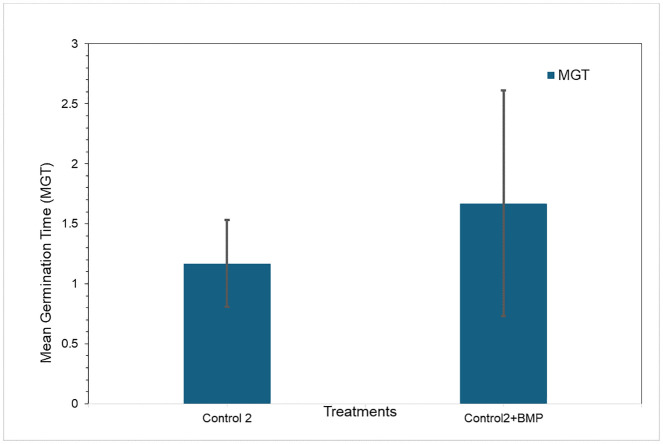
Effect of BMP on Mean germination time (MGT). The graph presents mean values and error bars present the standard deviation of the mean.

**Fig 7:**
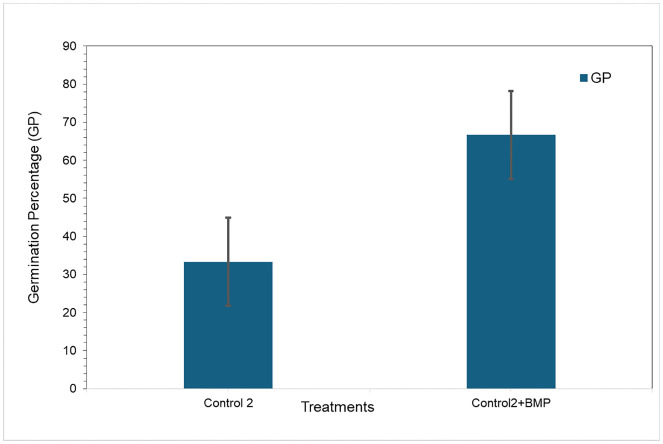
Effect of BMP on Germination percentage (GP). The graph presents mean values and error bars present the standard deviation of the mean.

**Fig 8:**
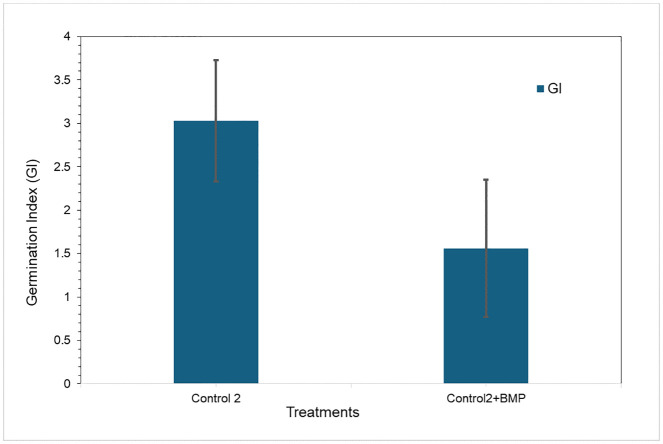
Effect of BMP on Germination index (GI). The graph presents mean values and error bars present the standard deviation of the mean.

Table 3 shows how BMP affected the germination parameters in the second experimental soil. There was no significant difference between the fertiliser treatment and the control. In the control soil, Malabar spinach seed germination started on third day and ended on sixth day; however, the application of BMP delayed both the start and end of germination (Table 3, Figs 4 and 5). The reduction in germination index, increase in germination percentage and mean germination time were noticed with BMP application. BMP increased the mean germination time of seeds by only 0.5 (Table 3, Fig 6), which is inversely related to the mean germination rate. BMP application had a significant impact on the germination percentage, which rose from 33.33% to 66.67% (Table 3, Fig 7). Neverthless, the addition of BMP resulted in a 1.47-unit decline in the germination index (Table 3, Fig 8).

**Table 3:** Germination parameters of Malabar spinach under second experimental condition.

Treatment	FDG	LDG	TSG	GP (%)	MGT (days)	MGR	GI
Control 2(T3)	3 (0)	5.33 (0.58)	2.33 (0.58)	33.33 (11.55)	1.17 (0.36)	0.89 (0.1)	3.03 (0.7)
Control 2 + BMP 1.5g(T4)	3.67 (0.58)	6.33 (1.15)	2.67 (0.58)	66.67 (11.55)	1.67 (0.94)	0.67 (0.16)	1.56 (0.79)

N.B. The values are mean (standard deviation) of three replications (Total sample size, N = 6; group size, n = 3 per treatment). Here control 1 = high EC soil; control 2 = low EC soil. The abbreviations: First Day of Germination (FDG), Last Day of Germination (LDG), Time Spread of Germination (TSG), Germination Percentage (GP), Mean Germination Time (MGT), Mean Germination Rate (MGR), Germination Index (GI).

BMP play a significant role in making available nutrients in the soil, but it cannot accelerate the seed germination process directly. Since germination is largely controlled by environmental factors such as moisture and temperature, the overall speed of germination can sometimes be slightly delayed [37]. Also, it can enhances seed viability and grow more seedlings due to containing slow-release phosphorus [8]. Consequently, a higher number of seeds successfully germinate. Therefore, although BMP does not necessarily shorten the mean germination time, it strengthen the germination percentage‌‌.

Meat and bone meal (MBM) can be a good substitute for natural, organic, and mineral nitrogen (N) and phosphorus (P) fertilizers that improves crop yields, soil fertility, and the quality of agricultural output [38]. In the study by Nogalska & Zaluszniewska the use of MBM without or with mineral nitrogen (N) on the experimental plot was a little bit greater than the unfertilized one [34].

### Impact of BMP on early growth parameters of Malabar Spinach

**Fig 9:**
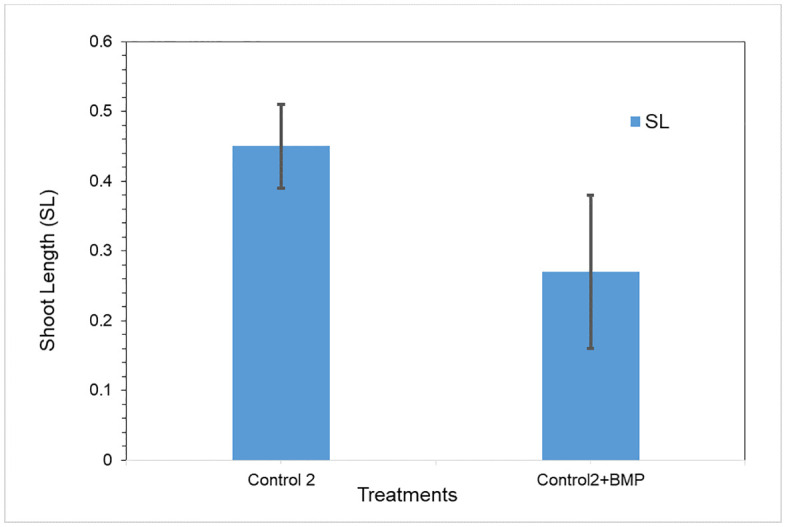
Effect of BMP on Shoot length (SL). The graph presents mean values and error bars present the standard deviation of the mean.

**Fig 10:**
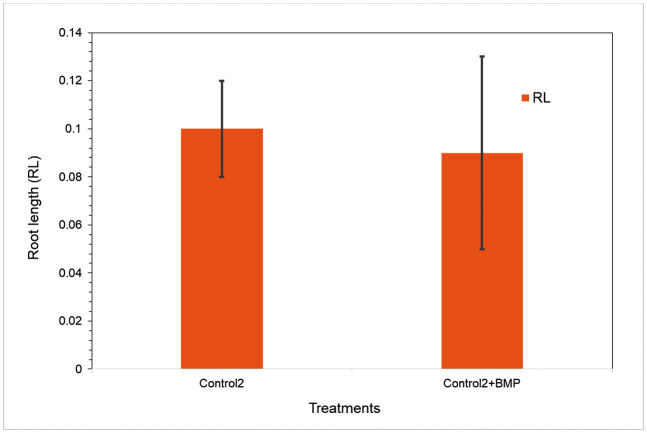
Effect of BMP on Root Length (RL). The graph presents mean values and error bars present the standard deviation of the mean.

**Fig 11:**
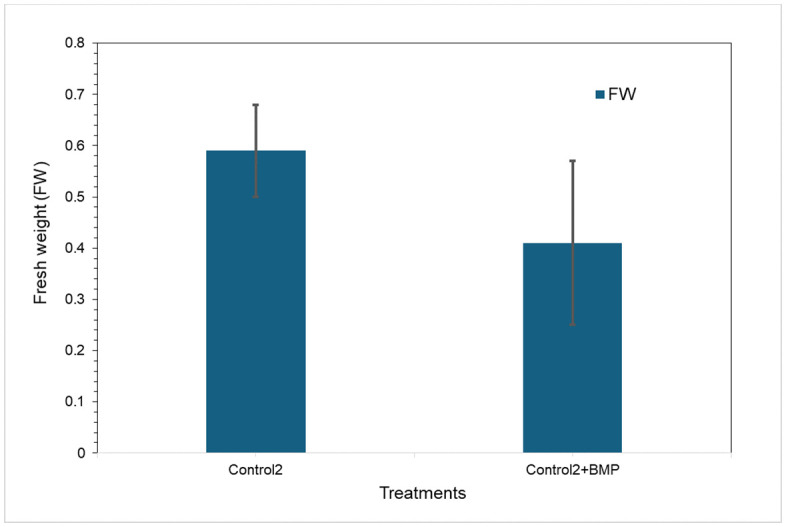
Effect of BMP on Fresh Weight (FW). The graph presents mean values and error bars present the standard deviation of the mean.

Results regarding the seedlings’ early growth (Table 4) revealed that bonemeal powder application to soil negatively influences. The effect of BMP on shoot length, root length, and fresh weight was not considerable in exp-2. Shoots were stronger in BMP (0.27 cm) compared to the control (Fig 9). Similarly, the root was slightly reduced in treated soil (0.09 cm) (Fig 10). Seedling treated with BMP decreased its fresh weight by 0.18g (Fig 11).

**Table 4:** t-test results for germination and early growth parameters under treatments (Experiment 2).

Two- sample t- test						
					95% Confidence Interval of the Difference	
**Germination Parameters**						
	**t**	**df**	**p-value**	**Mean difference**	**Lower**	**Upper**
**MGT**	−1.34	4	0.25	0.5	−1.53	0.53
**GP**	−3.54	4	0.02	33.33	−59.51	−7.16
**GI**	−0.14	4	0.90	0.05	−1.17	1.06
**Early Growth Parameters**						
**Shoot length**	2.66	4	0.06	0.19	−0.01	0.38
**Root length**	0.45	4	0.67	0.02	−0.07	0.09
**Fresh weight**	1.77	4	0.15	0.19	−0.10	0.48

N.B. Total sample size, N = 6; group size, n = 3 per treatment.

These results are in accordance with previous research that found that the root length and fresh weight of *Amaranthus cruentus* were negatively impacted by powdered chicken, mutton and beef bones whereas composted beef bone showed contrasting effect [39]. By the same token, Kivelä et al. investigated lower yields with MBM fertilization of sugar beet grown on clay loam and sandy clay soil gave 11.4% (2008) and 19.6% (2009) than mineral fertilizers [40]. The findings of mixing MBM with mineral NPK fertilizers were comparable to those of MBM alone in the same case. In contrast, Erdal demonstrated that bean seedlings treated with bone powder solutions (BPS), as an inorganic element source except Ca and P, increase root and shoot lengths as compared to their control and Calcium Phosphate (CP) treated seedlings [41]. Similarly, dry weight improvement of Bone powder solutions in wheat seedlings compared to the control was reported by Genise et al. [42]. The differences in the early growth parameters were further confirmed through t-test analysis (Table 4). Bone powder can be used as an alternative of synthesized chemical substances in agriculture and supply Ca and P. But its effect mechanism on plants is still not clear [42]. The application of BMP resulted in a greater reduction in shoot length, but only minor changes in root length and fresh weight were observed. The cause of this decline may be the slow breakdown of bone meal, which gradually releases phosphorus (P) needed for plant growth [43]. Similarly, longer roots may grow in the soil for the same reason. Overall, the findings show that early-stage seedling growth was not adversely affected by the application of BMP.

### Correlation analysis

(Figure in S3 File) presents the Pearson correlation among all the studied parameters. The Pearson correlation analysis revealed that mean germination time had strong negative correlation with root length (r = −0.91) and shoot length (r = −0.83), indicating statistically significant relationships (p < 0.05). This implied that as mean germination time increases the root and shoot length will decrease. In addition, mean germination time showed a moderate negative correlation with fresh weight (−0.57). Noli et al. reported that slower emergence was associated with lower emergence, resulting in smaller and more variable seedlings [44]. Slower germination in my study was associated with reduced seedling growth, including lower root length, shoot length, and fresh weight. Additionally, fresh weight showed a significant, strong and positive correlation with shoot length (r= + 0.88). However, the other parameters did not show significant correlations. A moderate correlation was observed for shoot length versus root length (r=+0.68), and root length versus fresh weight (r=+0.44), all of which were positive. Enhancement of root system increases the seedling’s water holding capacity [26] and facilitates greater uptake of water and dissolved nutrients, resulting in higher shoot length and fresh weight as well as plant growth and development [45]. Germination percentage showed negative correlations with shoot length, and fresh weight, with same correlation coefficients of −0.64. However, it did not show any correlation with root length (r = 0). It implied that while germination contributes somewhat to shoot growth, it has limited influence on root elongation. In contrast, germination percentage was positively correlated with mean germination time (r= + 0.29). There was a positive correlation between mean germination time and germination index (r = +0.55). Germination Index showed weak negative correlations with fresh weight (−0.43) and shoot length (−0.57), and strong association with root length (−0.77). A number of coefficients were developed to characterize the entire course of the seed germination process [46]. The higher the MGT, the slower a population of seeds reaches germination. The germination index is defined as a weighted sum of the daily numbers of germinated seeds. Consequently, the GI emphasizes both the percentage of germination and its speed. A higher GI value denotes a higher percentage and a higher rate of germination. However, these commonly used indices cannot fully distinguish different germination behaviors or capture every important feature of how seeds germinate [46]. The description supports my result of moderate association of germination index versus mean germination time and the correlation between germination index and germination percentage, showing nearly zero (+0.04). As a result, it occurs low seedlings growth.

## Conclusions

This study evaluated the impact of BMP as organic fertilizer on soil phosphorus availability, seed germination, and early growth performance of Malabar spinach. This investigation indicates that BMP increased soil pH, EC, and particularly phosphorus on both soil types, although repeated measurements should be carried out in this experiment. BMP showed the diminishing results of the germination parameters in experimental soil 2, because the study is conducted in a short period and BMP slowly releases nutrients including phosphorus. As a result, BMP did not improve the early growth parameters. However, minor differences in fresh weight and root length were observed, which may indicate a positive effect on plant growth over an extended period of time. Future research should measure other plant growth parameters, including plant biomass, shoot length, root length, leaf number, and phosphorus concentration in plant tissues to better evaluate nutrient uptake rather than only focusing on early growth. Similarly, yield parameters such as total biomass, leaf yield, and harvestable yield of Malabar spinach should be considered. Instead of working under pot conditions, field-scale experiments are needed to verify these findings and gain a better understanding of the impact of various BMP rates and frequencies on germination and plant growth under practical agricultural conditions.

## References

[1] Bakhat HF, Bibi N, Fahad S, Hammad HM, Natasha, Abbas S, et al. Rice husk bio-char improves brinjal growth, decreases insect infestation by enhancing silicon uptake. Silicon. 2020;13(10):3351–60. doi: 10.1007/s12633-020-00719-4

[2] Upadhyay V, Choudhary K, Agrawal S. Use of biochar as a sustainable agronomic tool, its limitations and impact on environment: a review. Discov Agric. 2024;2.

[3] Egnime KK, Outéndé T, Koffi BA. Influence of reasoned organic and inorganic fertilization on okra (Abelmoschus esculentus) growth, productivity, and profitability on degraded sandy soil in South Togo. Discov Agric. 2023;1.

[4] Pahalvi HN, Rafiya L, Rashid S, Nisar B, Kamili AN. Chemical fertilizers and their impact on soil health. In: Dar GH, Bhat RA, Mehmood MA, Hakeem KR, editors. Microbiota and Biofertilizers, Vol 2. Cham: Springer International Publishing; 2021. pp. 1–20. doi: 10.1007/978-3-030-61010-4_1

[5] Kabir K, Mukut M, Rahman S, Chowdhury A, Mukta M, Asfour M. Farmers’ perceptions and capacity for 3Rs agro-waste management in a vegetable growing area of Bangladesh. Discov Agric. 2023;1.

[6] Stępień A, Wojtkowiak K, Kolankowska E. Use of meat industry waste in the form of meat-and-bone meal in fertilising maize (Zea mays L.) for grain. Sustain. 2021;13:2857.

[7] Mohammed E, Tewodros M, Lulseged T, Feyera L, Wuletawu A, Amsalu T. Agronomic and socio-economic drivers of fertilizer use and crop productivity in smallholder wheat production systems in Ethiopia. Discov Environ. 2024;2(1):130. doi: 10.1007/s44274-024-00162-x 39563662 PMC11575296

[8] Minson DJ. Phosphorus. Forage in Ruminant Nutrition. San Diego and Sydney: Academic Press (Elsevier); 1990. pp. 230–64.

[9] Jeng AS, Haraldsen TK, Pedersen AG, P A. Meat and bone meal as nitrogen and phosphorus fertilizer to cereals and rye grass. Nutr Cycl Agroecosyst. 2006;76:183–91.

[10] Sims JT, Sharpley AN. Phosphorus and Plant Nutrition: An Overview. In: Phosphorus: Agriculture and the Environment. Madison: American Society of Agronomy; Crop Science Society of America; Soil Science Society of America.; 2005. pp. 353–78.

[11] Zwetsloot MJ, Lehmann J, Bauerle T, Vanek S, Hestrin R, Nigussie A. Phosphorus availability from bone char in a P-fixing soil influenced by root-mycorrhizae-biochar interactions. Plant Soil. 2016;408(1–2):95–105. doi: 10.1007/s11104-016-2905-2

[12] Zimmer D, Kruse J, Siebers N, Panten K, Oelschläger C, Warkentin M, et al. Bone char vs. S-enriched bone char: Multi-method characterization of bone chars and their transformation in soil. Sci Total Environ. 2018;643:145–56. doi: 10.1016/j.scitotenv.2018.06.076 29936158

[13] Załuszniewska A, Nogalska A. The effect of meat and bone meal (MBM) on phosphorus (P) content and uptake by crops, and soil available P balance in a six-year field experiment. Sustainability. 2022;14(5):2855. doi: 10.3390/su14052855

[14] Pan M, Yau Pc, Lee Kc, Man HY. Effects of different fertilizers on the germination of tomato and cucumber seeds. Water Air Soil Pollut. 2022;233(1). doi: 10.1007/s11270-021-05494-5

[15] Maggio A, Raimondi G, Martino A, Pascale SDE. Salt stress response in tomato beyond the salinity tolerance threshold. Env Exp Bot. 2007;59:276–82.

[16] Gomez K, Gomez A. Completely randomized design. Statistical procedures for agricultural research. 2 ed. New York: John Wiley & Sons; 1984. pp. 20–30.

[17] Juo ASR. Selected methods for soil and plant analysis [Internet]. Ibadan; 1978. Available from: https://cgspace.cgiar.org/items/8360b1fa-6d72-41c6-8d45-b9ba33255c65

[18] Spiteri K, Sacco AT. Estimating the electrical conductivity of a saturated soil paste extract (EC e) from 1:1(EC 1:1), 1:2(EC 1:2) and 1:5(EC 1:5) soil:water suspension ratios, in calcareous soils from the Mediterranean Islands of Malta. Commun Soil Sci Plant Anal. 2024;55(9):1302–12. doi: 10.1080/00103624.2024.2304636

[19] Kibria K, Islam MdA, Hossain MZ, Billah SM. Calibration of yellow colour spectroscopic method of phosphorus determination for wavelength, working range and time. Khulna Univ Stud. 2019;:41–6. doi: 10.53808/kus.2019.16.1and2.1901-l

[20] Adilu G, Gebre Y. Effect of salinity on seed germination of some tomato (Lycopersicon esculentum Mill.) varieties. J Arid Agric. 2021.

[21] Ali L, Xiukang W, Naveed M, Ashraf S, Nadeem SM, Haider FU, et al. Impact of biochar application on germination behavior and early growth of maize seedlings: insights from a growth room experiment. Appl Sci. 2021.

[22] Kader MA. A comparison of seed germination calculation formulae and the associated interpretation of resulting data. J Proc R Soc New South Wales. 2005;138(3–4):65–75. doi: 10.5962/p.361564

[23] Ranmeechai N, Lacap A, Tac-an MI, Bayogan ERV. Seed germination and vigor of four philippine rice varieties as influenced by hydropriming and storage at various durations. Philipp J Sci. 2022.

[24] Deydier E, Guilet R, Sharrock P. Beneficial use of meat and bone meal combustion residue: “an efficient low cost material to remove lead from aqueous effluent”. J Hazard Mater. 2003;101(1):55–64. doi: 10.1016/s0304-3894(03)00137-7 12850320

[25] Atuah L, Hodson ME. A comparison of the relative toxicity of bone meal and other P sources used as remedial treatments to the earthworm Eisenia fetida. Pedobiologia. 2011;54:S181–6. doi: 10.1016/j.pedobi.2011.07.009

[26] Asare W. Effects of bone meal on physiochemical soil properties of a fertilized reclamation site in Iceland [Internet]. United Nations University Land Restoration Training Programme. 2019. Available from: https://www.grocentre.is/static/gro/publication/736/document/asare2019.pdf

[27] Liu X, Li X, Hua Y, Sinkkonen A, Romantschuk M, Lv Y, et al. Meat and bone meal stimulates microbial diversity and suppresses plant pathogens in asparagus straw composting. Front Microbiol. 2022;13:953783. doi: 10.3389/fmicb.2022.953783 36204619 PMC9530395

[28] Ylivainio K, Turtola RU. Meat bone meal and fox manure as P sources for ryegrass (Lolium multiflorum) grown on a limed soil. Nutr Cycl Agroecosyst. 2008;81:267–78.

[29] Nogalska A, Zalewska M. The effect of meat and bone meal on phosphorus concentrations in soil and crop plants. Plant Soil Environ. 2013;59(12):575–80. doi: 10.17221/594/2013-pse

[30] Pel R, Dupin S, Schat H, Ellers J, Kiers ET, van Straalen NM. Growth benefits provided by different arbuscular mycorrhizal fungi to Plantago lanceolata depend on the form of available phosphorus. Eur J Soil Biol. 2018;88:89–96. doi: 10.1016/j.ejsobi.2018.07.004

[31] García-Díaz C, Siles JA, Bastida F, Moreno JL. Soil characteristics rather than microbial inoculation determines the effects of alternative phosphorus-fertilizers on ryegrass yield and the soil microbial community. J Soil Sci Plant Nutr. 2025;25(2):2420–37. doi: 10.1007/s42729-025-02275-5

[32] Azeez J, Bankole G, Aghorunse A, Odelana T, Oguntade O. Evaluating the environmental and agronomic implications of bone char and biochar applications to loamy sand based on sorption data. Environ Syst Res. 2024;13.

[33] Khan F, Siddique AB, Shabala S, Zhou M, Zhao C. Phosphorus plays key roles in regulating plants’ physiological responses to abiotic stresses. Plants (Basel). 2023;12(15):2861. doi: 10.3390/plants12152861 37571014 PMC10421280

[34] Nogalska A, Załuszniewska A. The effect of meat and bone meal applied without or with mineral nitrogen on macronutrient content and uptake by winter oilseed rape. J Elem. 2020.

[35] Kim K-S, Yoo J-S, Kim S, Lee HJ, Ahn K-H, Kim IS. Relationship between the electric conductivity and phosphorus concentration variations in an enhanced biological nutrient removal process. Water Sci Technol. 2007;55(1–2):203–8. doi: 10.2166/wst.2007.053 17305141

[36] Mondini C, Cayuela ML, Sinicco T, Sánchez-Monedero MA, Bertolone E, Bardi L. Soil application of meat and bone meal. Short-term effects on mineralization dynamics and soil biochemical and microbiological properties. Soil Biol Biochem. 2008;40(2):462–74. doi: 10.1016/j.soilbio.2007.09.010

[37] Khaeim H, Kende Z, Jolánkai M, Kovács GP, Gyuricza C, Tarnawa Á. Impact of temperature and water on seed germination and seedling growth of Maize (Zea mays L.). Agronomy. 2022;12(2):397. doi: 10.3390/agronomy12020397

[38] Nogalska A, Załuszniewska A. The effect of meat and bone meal (MBM) on crop yields, nitrogen content and uptake, and soil mineral nitrogen balance. Agronomy. 2021;11(11):2307. doi: 10.3390/agronomy11112307

[39] Yasmin D, Khan MdZ, Billah SM. Effects of composted and powdered bones meal on the growth and yield of Amaranthus cruentus. Asian J Res Crop Sci. 2018;2(3):1–9. doi: 10.9734/ajrcs/2018/45241

[40] Kivelä J, Chen L, Muurinen S, Kivijärvi P, Hintikainen V, Helenius J. Effects of meat bone meal as fertilizer on yield and quality of sugar beet and carrot. Agric Food Sci. 2015;24(2):68–83. doi: 10.23986/afsci.8587

[41] Erdal S. Comparative evaluation of the effects of bone powder and calcium phosphate on plant growth and development. Phosphorus Sulfur Silicon Relat Elem. 2012;187(9):1017–25. doi: 10.1080/10426507.2012.658982

[42] Genisel M, Erdal S, Turk H, Dumlupinar R. The availability of bone powder as inorganic element source on growth and development in wheat seedlings. Toxicol Ind Health. 2012;28(5):458–62. doi: 10.1177/0748233711414610 21937527

[43] Shaji H, Chandran V, Mathew L. Organic fertilizers as a route to controlled release of nutrients. In: Allam Z, Chabaud D, Moreno C, editors. Controlled release fertilizers for sustainable agriculture. Amsterdam: Academic Press (Elsevier); 2021. pp. 231–45.

[44] Noli E, Beltrami E, Casarini E, Urso G, Conti S. Reliability of early and final counts in cold and cool germination tests for predicting maize seed vigour. Ital J Agron. 2010;5:383–91.

[45] Sasi S, Prakash P, Hayden S, Dooley D, Poiré R, Hu T, et al. Enhanced plant growth on simulated martian regolith via water chemistry optimisation: the role of RONS and Nano/Micro-bubbles. Int J Mol Sci. 2025;26(17):8318. doi: 10.3390/ijms26178318 40943238 PMC12428210

[46] Talská R, Machalová J, Smýkal P, Hron K. A comparison of seed germination coefficients using functional regression. Appl Plant Sci. 2020;8(8):e11366. doi: 10.1002/aps3.11366 32995101 PMC7507017

